# Metabolic reprogramming contributes to radioprotection by protein kinase Cδ

**DOI:** 10.1016/j.jbc.2023.105186

**Published:** 2023-08-21

**Authors:** Angela M. Ohm, Trisiani Affandi, Julie A. Reisz, M. Cecilia Caino, Angelo D’Alessandro, Mary E. Reyland

**Affiliations:** 1 Department of Craniofacial Biology, School of Dental Medicine, University of Colorado Anschutz Medical Campus, Aurora, Colorado, USA; 2 Department of Biochemistry and Molecular Genetics, University of Colorado Anschutz Medical Campus, Aurora, Colorado, USA; 3 Department of Pharmacology, School of Medicine, University of Colorado Anschutz Medical Campus, Aurora, Colorado, USA

**Keywords:** protein kinase Cδ, metabolism, radioprotection, salivary gland, apoptosis

## Abstract

Loss of protein kinase Cδ (PKCδ) activity renders cells resistant to DNA damaging agents, including irradiation; however, the mechanism(s) underlying resistance is poorly understood. Here, we have asked if metabolic reprogramming by PKCδ contributes to radioprotection. Analysis of global metabolomics showed that depletion of PKCδ affects metabolic pathways that control energy production and antioxidant, nucleotide, and amino acid biosynthesis. Increased NADPH and nucleotide production in PKCδ-depleted cells is associated with upregulation of the pentose phosphate pathway (PPP) as evidenced by increased activation of G6PD and an increase in the nucleotide precursor, 5-phosphoribosyl-1-pyrophosphate. Stable isotope tracing with U-[^13^C_6_] glucose showed reduced utilization of glucose for glycolysis in PKCδ-depleted cells and no increase in U-[^13^C_6_] glucose incorporation into purines or pyrimidines. In contrast, isotope tracing with [^13^C_5_, ^15^N_2_] glutamine showed increased utilization of glutamine for synthesis of nucleotides, glutathione, and tricarboxylic acid intermediates and increased incorporation of labeled glutamine into pyruvate and lactate. Using a glycolytic rate assay, we confirmed that anaerobic glycolysis is increased in PKCδ-depleted cells; this was accompanied by a reduction in oxidative phosphorylation, as assayed using a mitochondrial stress assay. Importantly, pretreatment of cells with specific inhibitors of the PPP or glutaminase prior to irradiation reversed radioprotection in PKCδ-depleted cells, indicating that these cells have acquired codependency on the PPP and glutamine for survival. Our studies demonstrate that metabolic reprogramming to increase utilization of glutamine and nucleotide synthesis contributes to radioprotection in the context of PKCδ inhibition.

Many diseases are associated with disruption of normal metabolism, and metabolic regulators are emerging as druggable targets or cotargets for cancer therapy (1, 2). Protein kinase Cδ (PKCδ) is a broadly functional, ubiquitous protein kinase that regulates a variety of cellular processes including proliferation, survival, and cell death (3). For example, we have shown that PKCδ is required for DNA damage–induced apoptosis, and genetic ablation or inhibition of PKCδ is protective against irradiation (IR) and other types of DNA damage (4, 5, 6, 7). Likewise, loss of PKCδ function *in vivo* is protective in many mouse models of tissue injury (3). The diverse functions of PKCδ may derive in part from its ability to modulate fundamental homeostatic mechanisms such as metabolism.

While the DNA damage response (DDR) is typically thought of as a series of nuclear events that protect DNA integrity, extranuclear responses to DNA damage are also clearly important for cell survival. These can include metabolic reprogramming to increase generation of nucleotides for DNA synthesis and/or DNA repair, activation of antioxidant pathways, and alterations in pathway utilization for energy production (8). The contribution of PKCδ to cell death *via* extranuclear signaling is not well understood; however, there is evidence that PKCδ can regulate some mitochondrial functions. For example, PKCδ regulates energy flux at the mitochondria, which plays a central role in executing cell death programs *via cytochrome c* release and pro-apoptotic signal activation (9, 10, 11). In addition, PKCδ promotes apoptosis by modulating mitochondrial ROS production (12, 13), which in turn activates PKCδ (14).

Here, we have explored a mechanistic connection between PKCδ, metabolic reprogramming, and IR-induced cell death. Our studies show that PKCδ knockdown (KD) results in dramatic alterations in metabolism resulting in the suppression of oxidative phosphorylation, increased glycolysis, and increased use of glutamine for biosynthetic pathways. PKCδ KD also results in activation of the pentose phosphate pathway (PPP) for generation of NADPH, a cofactor essential to redox homeostasis, and increased production of nucleotides. Importantly, this metabolic reprogramming is necessary for resistance to cell death upon depletion or inhibition of PKCδ. Our studies define a novel mechanism for regulation of IR-induced cell death by PKCδ and suggest potential new targets for radioprotection.

## Results

### PKCδ regulates metabolic pathways that control energy production, nucleotide, antioxidant, and amino acid biosynthesis

**Figure 1:**
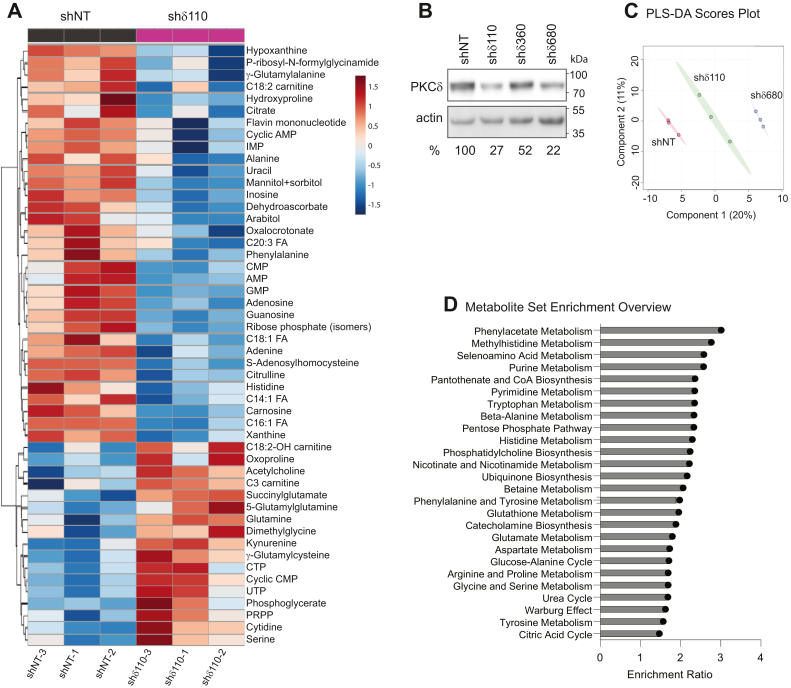
**Global metabolic profiling of Par-C5 cells depleted of PKCδ (shδ110) or untargeted control shRNA (shNT).** Untargeted metabolomics was performed on triplicate biological samples of Par-C5 cells depleted of PKCδ (shδ110) or untargeted control shRNA (shNT). *A*, heat map with hierarchical clustering of the top 50 significant metabolites in Par-C5 shNT and shδ110 cells is shown following student’s *t* test analysis (α = 0.05). *B*, immunoblot showing the depletion of PKCδ with three different shRNA constructs. Densitometry quantifying the percent of PKCδ expression, as compared to shNT and normalized to actin, is shown below the blots. *C*, partial least squares discriminant analysis (PLS-DA) scores plot of metabolomics data. Shaded ovals represent 95% confidence intervals. *D*, enrichment analysis of shδ110 cells as compared to shNT cells. The top 26 metabolite groups from quantitative enrichment analysis are shown in the bar chart (*p* < 0.05). Enrichment ratio represents the number of observed metabolite hits divided by the number of expected metabolite hits within each pathway. PKCδ, protein kinase Cδ.

We hypothesized that regulation of metabolic functions could contribute to the wide-ranging effects of PKCδ on proliferation, cell death, and DNA repair. To test this hypothesis, we performed global metabolomics on Par-C5 salivary acinar cells engineered to express a nontargeted shRNA control (shNT) or two unique shRNAs targeted to PKCδ (shδ110 and shδ680). Figure 1*B* shows depletion of PKCδ in all Par-C5 shδ cell lines used in this manuscript. A heat map view of the top 50 metabolites (by *t* test *p*-values) in shNT and shδ110 cells reveals that PKCδ KD globally alters steady state levels of metabolites (Fig. 1*A*), an observation also supported by partial least squares discriminant analysis (PLS-DA) whereby PKCδ status clusters along principal component 1, which explains 38.3% of the phenotypic variance between groups (component 1, Fig. 1*C*). Analogous data comparing Par-C5 shNT and shδ680 cell metabolites is shown in Fig. S1, *A* and *B* and in a second independent cell line, A549 (Fig. S2, *A*–*C*). A549 human lung cancer cells, like Par-C5 cells, require PKCδ for cell death, and depletion of PKCδ increases survival in A549 cells treated with DNA damaging agents (15).

**Figure 2:**
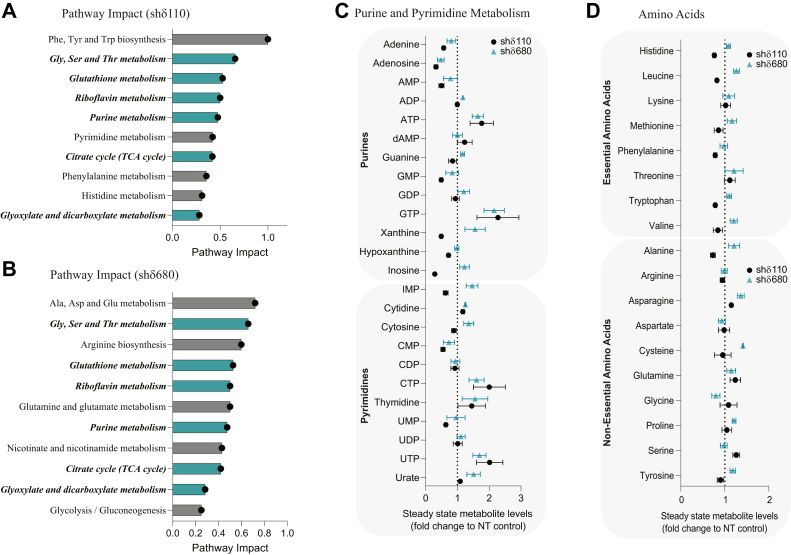
**Metabolic pathways impacted by loss of PKCδ.***A* and *B*, pathway impact (>1.2 fold compared to shNT cells, *p* < 0.1) was determined by comparing untargeted global metabolomics of Par-C5 cells depleted of PKCδ shδ110 (*A*) and shδ680 (*B*) cells to control cells (shNT). Pathway impact quantifies the number of metabolites that are present in a particular pathway following enrichment and pathway topology analysis. Shown are pathways with greater than 0.25 pathway impact score. Italicized and bolded pathway labels with *teal* bars are those shared between shδ110 and shδ680 depleted cells. *C* and *D*, steady state levels of individual metabolites from the nucleotide and amino acid pathways shown in *A* and *B*. Data is presented as fold change of shδ110 cells (*black circle*) or shδ680 cells (*teal triangle*) relative to shNT control. Error bars represent SEM from a representative experiment with triplicate biological replicates. PKCδ, protein kinase Cδ.

Enrichment over-representation analysis revealed that metabolites in 26 pathways were significantly enriched in shδ110 cells (Fig. 1*D*). Eleven (42%) of these pathways were also significant in shδ680 cells (Fig. S1*B*). Together, these commonly disrupted pathways include nucleotide and amino acid metabolism, the tricarboxylic acid (TCA) cycle, the Warburg effect, and glutathione metabolism. Pathway impact analysis was then performed on significantly altered metabolites (*p* < 0.1, 1.2-fold change cutoff) from Par-C5 shδ110 and shδ680 cells (Fig. 2, *A* and *B*). Quantification of the pathway impact factor involves determining the number of metabolites that exist within the pathway after conducting an analysis of its topology and enrichment (16). Comparison of Par-C5 shNT cells to shδ110 and shδ680 cells shows that 6 of 13 pathways impacted are shared between both PKCδ KD cell lines (Fig. 2, *A* and *B*, bold and italicized). Top pathways impacted include purine, riboflavin, and glutathione metabolism, the TCA cycle, and amino acid metabolism. Notably, the PPP and pyrimidine pathways were significantly impacted in shδ110 cells (Fig. 2*A*) and in both A459 shδ cell lines (Fig. S2, *D* and *E*), while purine metabolism was impacted in all Par-C5 and A549 shδ cell lines. Likewise, the nicotinate/nicotinamide pathway was significantly impacted in both A549 shδ cell lines (Fig. S2, *D* and *E*) and in Par-C5 shδ680 cells (Fig. 2*B*).

The relative abundance of metabolites in nucleotide and amino acid metabolism are shown in Figure 2, *C* and *D*. Purine and pyrimidine triphosphate nucleotides (ATP, GTP, CTP, and UTP) are increased in both Par-C5 shδ110 and shδ680 cells compared to shNT cells (Fig. 2*C*). Similar changes are seen in two A549 shδ cells (Fig. S3*A*). Changes in the steady state abundance of specific amino acids are shown in Figure 2*D*. While many amino acids showed increased abundance in shδ680 cells, only threonine, asparagine, and glutamine were increased in both cell lines. Taken together, this indicates that PKCδ plays a role in regulating the steady state level of molecules needed for proliferation (amino acids and nucleotides), redox homeostasis (glutathione, nicotinamide), and energy production (TCA cycle and glycolysis).

### PKCδ-depleted cells upregulate the PPP

**Figure 3:**
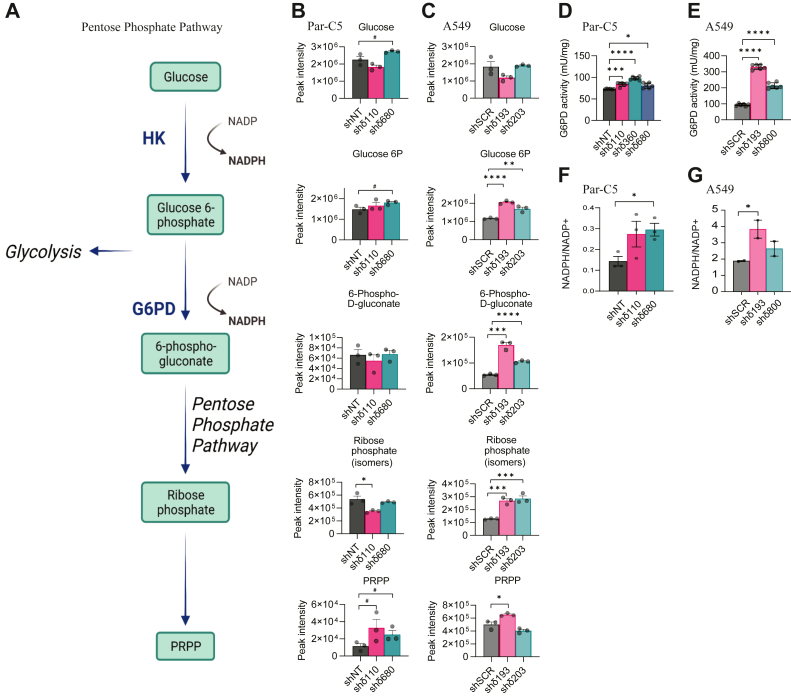
**Linkage of PKCδ status to the pentose phosphate pathway.** Global metabolomic analysis was performed on either Par-C5 cells depleted of PKCδ (shδ110 or shδ680) or untargeted control shRNA (shNT) or A549 cells depleted of PKCδ (shδ193 or shδ800) or untargeted control shRNA (shSCR). *A*, schematic of pentose phosphate pathway (PPP). *B* and *C*, quantification of selected PPP metabolites in Par-C5 cells (*B*) and A549 cells depleted of PKCδ (*C*). *D* and *E*, G6PD activity in Par-C5 and A549 PKCδ depleted cells. *F* and *G*, NADPH/NADP+ ratio was determined for Par-C5 and A549 PKCδ depleted cells. G6PD and NADPH+/NADP+ experiments were repeated a minimum of three times with triplicate biological replicates. Data represents the average of a minimum of n = 3 experiments. Error bars represent SEM, statistics represent 1-way ANOVA, #*p* < 0.10, ∗*p* < 0.05, ∗∗*p* < 0.01, ∗∗∗*p* < 0.001, ∗∗∗∗*p* < 0.0001. G6PD, glucose 6-phosphate dehydrogenase; PKCδ, protein kinase Cδ.

Shuttling of glucose through the PPP results in generation of reducing equivalents in the form of NADPH along with ribose sugars to produce nucleobases for proliferation and DNA repair. Based on our data that depletion of PKCδ increases nucleotide synthesis, we analyzed the abundance of metabolites in the PPP pathway in Par-C5 and A549 PKCδ depleted cells (Fig. 3, *A*–*C*). Our data shows that steady state glucose levels do not change significantly with PKCδ KD, while glucose 6-phosphate (G6P) is slightly increased in Par-C5 cells and more dramatically increased in A549 cells depleted of PKCδ (Fig. 3, *B* and *C*). An increase in steady state levels of PPP intermediates can be explained either by increased biosynthesis or less consumption of the intermediates for ancillary pathways. Thus, we examined the enzymatic activity of glucose 6-phosphate dehydrogenase (G6PD), the enzyme that converts G6P to 6-phosphogluconolactone and the rate limiting enzyme for the PPP. We found that G6PD enzymatic activity is increased up to 1.3-fold in three Par-C5 shδ cell lines and up to 3.5-fold in two A549 cell lines that express shδ (Fig. 3, *D* and *E*). In addition, shδ cells showed increased steady state levels of 6-phosphogluconate and ribose phosphate (A549 shδ cells) and glutamine 5-phosphoribosyl-1-pyrophosphate (PRPP) amidotransferase, the rate-limited step in *de novo* purine synthesis. Additional PPP metabolites were elevated in A549 cells (Fig. S3*B*) but were below the level of detection in Par-C5 cells. This suggests that A549 cells (human lung cancer) maintain greater pools of PPP intermediates compared to Par-C5 cells (rat epithelial cells) upon PKCδ depletion.

Increased utilization of the PPP is consistent with an increased supply of nucleotides for DNA repair. The PPP is also the major source of NADPH, which provides reducing power for generation of nucleotides and other biosynthetic pathways. We next assayed steady state levels of NADP+ and NADPH in Par-C5 and A549 cells depleted of PKCδ. The ratio of NADPH:NADP+ was increased up to two fold in shδ Par-C5 cells and A549 cells (Fig. 3, *F* and *G*). Note that nicotinate and nicotinamide metabolites, precursors to NAD, are also enriched in Par-C5 shδ110 cells (Fig. 1*C*) and this pathway was among the top four pathways impacted in A549 shδ193 and shδ203 cells (Fig. S2, *C* and *D*).

### PKCδ-depleted cells have reduced utilization of glucose and increased utilization of glutamine

**Figure 4:**
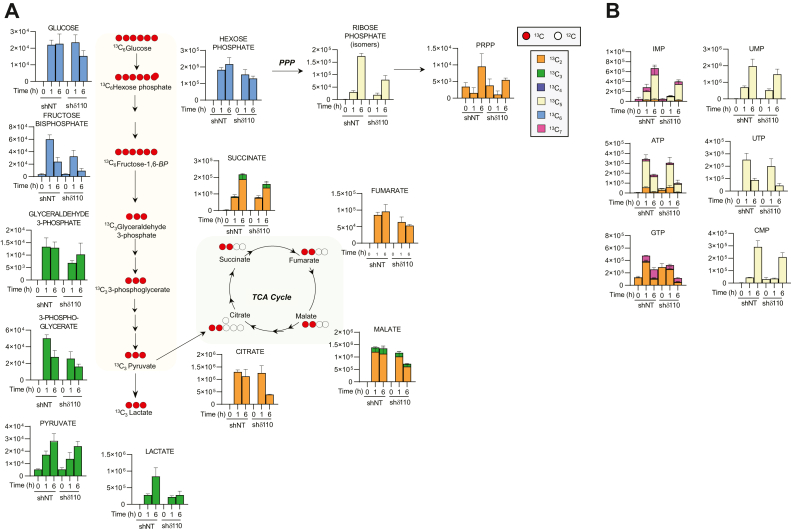
**Stable isotope tracing in Par-C5 shδ110 cells with U-**^**13**^**C glucose.** Par-C5 cells were starved of glucose for 18 h and then labeled with complete media substituted with labeled [U-^13^C]glucose for 0, 1, and 6 h. *A*, schematic showing glycolysis, pentose phosphate pathway (PPP), and TCA cycle metabolites following addition of labeled glucose. *Top* legend indicates labels for ^13^C (*red circle*) or ^12^C (*white circle*) in glycolysis, TCA, and PPP schematic. Multi-colored legend (boxes) shows ^13^C isotopologs for all tracing graphs. *B*, ^13^C enrichment in nucleotides; TCA, tricarboxylic acid.

To determine how PKCδ alters glucose utilization, we performed stable isotope tracing with uniformly labeled U-[^13^C_6_] glucose. Par-C5 shNT and shδ110 cells were starved for glucose 18 h prior to the addition of the metabolic tracer; metabolite ^13^C enrichment was assayed at 0, 1, and 6 h (Fig. 4*A*). Compared to Par-C5 shNT (control) cells, shδ110 cells showed a modest decrease in uptake of [^13^C_6_] glucose at 6 h. Incorporation of ^13^C atoms into glycolytic metabolites downstream of glucose, and in TCA cycle metabolites, was likewise decreased at the six-hour time point in shδ110 cells.

Given the reduced utilization of [^13^C_6_] glucose for glycolysis and the increase in utilization of the PPP as shown in the global metabolic analysis (Fig. 3), we asked if glucose is preferentially being used for nucleotide synthesis. Figure 4*A* shows that ^13^C enrichment in ribose phosphate and PRPP is reduced in Par-C5 shδ110 cells compared to shNT cells. Likewise, utilization of [^13^C_6_] glucose for both purine and pyrimidine nucleotide synthesis *via* the PPP (yellow bar) is reduced (Fig. 4*B*). However, we observed that the steady state level of ^12^C nucleotides is increased in Par-C5 shδ cells (“zero” time point Fig. S4*A*), supporting our global metabolomics analysis (Fig. 2*C*) that shows overall increased nucleotide synthesis in shδ cells. Based on these findings, we conclude that increased nucleotide synthesis is not driven by increased utilization of glucose.

**Figure 5:**
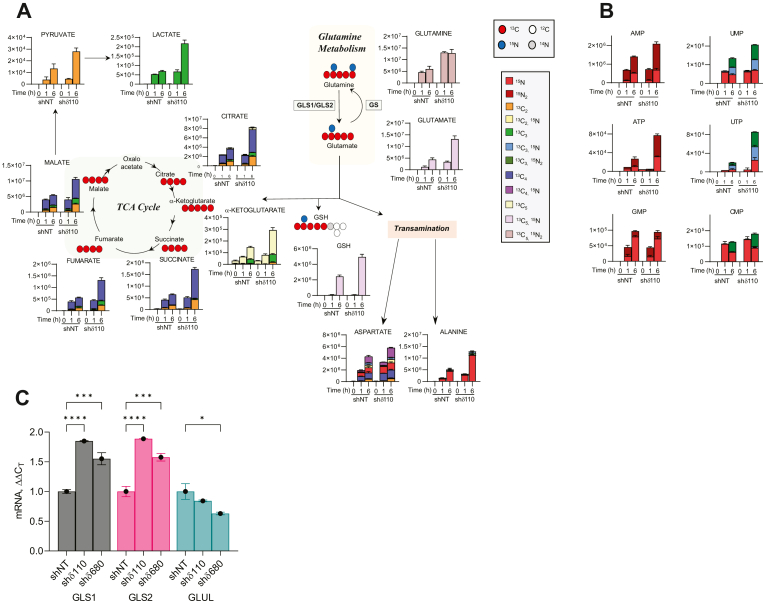
**Stable isotope tracing in Par-C5 shδ110 cells with [**^**13**^**C**_**5**_**,**^**15**^**N**_**2**_**] glutamine.** Par-C5 cells were starved of glutamine for 18h and then incubated with complete media substituted with labeled glutamine for 0, 1, and 6 h. *A*, schematic of relevant glutamine metabolism pathways. *Top* legend indicates labels for ^13^C (*red circle*), ^12^C (*white circle*), ^15^N (*blue circle*), or ^14^N (*gray circle*) in glutamine pathway schematic. Multi-colored legend (boxes) shows ^13^C^15^N isotopologs for all tracing graphs. *B*, ^13^C,^15^N enrichment in nucleotides. *C*, q-RT PCR for GLS1, GLS2, and GLUL was performed on Par-C5 cells depleted of PKCδ (shδ110 or shδ680) or untargeted control shRNA (shNT). Graph represents fold change in mRNA expression (ΔΔC_T_) as compared to shNT = 1. A representative experiment, including triplicate samples, is shown. q-RT PCR experiments were repeated a minimum of three times. Error bars represent SEM, statistics represent 1-way ANOVA, ∗*p* < 0.05, ∗∗∗*p* < 0.001, ∗∗∗∗*p* < 0.0001. GLS1, glutaminase-1; GLS2, glutaminase-2; GLUL, glutamine synthetase; PKCδ, protein kinase Cδ.

Glutamine is a likely source of alternative fuel in PKCδ-depleted Par-C5 cells as it can drive the TCA cycle, amino acid, nucleotide, and antioxidant biosynthesis, all of which are upregulated upon depletion of PKCδ (Figs. 1 and 2). Analysis of [^13^C_5_,^15^N_2_] glutamine flux shows that both glutamine uptake and conversion of glutamine to glutamate are increased in PKCδ-depleted cells (Fig. 5*A*). PKCδ KD increases utilization of glutamine for the TCA cycle as judged in particular by elevated levels of ^13^C_5_ α-ketoglutarate and ^13^C_4_ levels of subsequent TCA intermediates including succinate, fumarate, malate, and citrate (Fig. 5*A*). Further, in PKCδ-depleted cells, glutamate is also used *via* transamination to produce aspartate and alanine (from oxaloacetate and pyruvate, respectively) and for synthesis of glutathione (GSH, Fig. 5*A*). Notably, in Par-C5 shδ cells, increased utilization of glutamine is accompanied by increased expression of glutaminase 1/2, the enzyme which converts glutamine to glutamate, and a modest reduction in glutamine synthetase (GLUL) which catalyzes the reverse reaction (Fig. 5*C*).

We then asked if glutamine is preferentially being used for nucleotide synthesis. [^13^C_5_, ^15^N_2_] glutamine can contribute to purine synthesis through donation of ^15^N. Figure 5*B* shows that incorporation of ^15^N from glutamine into both AMP and ATP, but not GMP, is increased dramatically in Par-C5 shδ cells. [^13^C_5_,^15^N_2_] glutamine can also contribute to pyrimidine synthesis *via* donation of ^15^N or through donation of ^13^C_3_ and ^15^N from aspartate. As shown in Figure 5*B*, synthesis of UMP from [^13^C_5_,^15^N_2_] glutamine directly (red bars and green bars) and *via* aspartate (blue bars and green bars) is also increased dramatically in Par-C5 shδ cells. Likewise, utilization of [^13^C_5_,^15^N_2_] glutamine for synthesis of CMP (*via* UMP) is increased (Fig. 5*B*). Taken together, our data indicates that in the absence of PKCδ, Par-C5 cells increase utilization of glutamine for nucleotide synthesis. Increased production of ribose phosphate from elevated flux through the PPP may also contribute to nucleoside synthesis, complementing glutaminolysis dependence for synthesis of purine and pyrimidine bases.

### PKCδ depletion suppresses oxidative phosphorylation and increases anaerobic glycolysis

**Figure 6:**
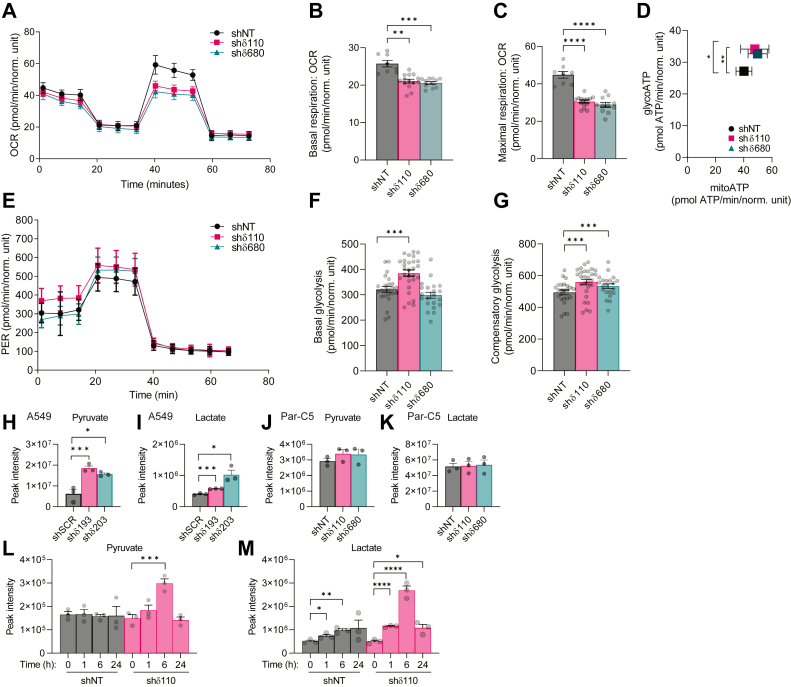
**Depletion of PKCδ suppresses oxidative phosphorylation and increases glycolysis.***A*, Agilent Seahorse Mito Stress test was used to assay mitochondrial oxygen consumption rates in Par-C5 control (shNT) and PKCδ-depleted cells (shδ110 and shδ680). Graph shows oxygen consumption rate (OCR, pmol/min/norm. unit) of cells over time when treated sequentially with 1.5 μM oligomycin, 1 μM FCCP, and 0.5 μM rotenone/antimycin. From the tracing in (*A*), OCR-associated measurements for basal respiration (*B*) and maximal respiration (*C*) were calculated using Agilent Seahorse Analytics software. *D*, Agilent Seahorse ATP Rate assay was used to quantify the contribution of either mitochondrial (mitoATP) or glycolytic (glycoATP) ATP production rates in Par-C5 control (shNT) and PKCδ-depleted cells (shδ110 and shδ680). *E*, Agilent Seahorse Glycolytic Rate Assay shows proton efflux rate (PER) in cells treated sequentially with 0.5 μM rotenone/antimycin A and 50 mM 2-deoxyglucose. From the tracing in (*E*), basal glycolysis (*F*) and compensatory glycolysis (*G*) were calculated with Agilent Seahorse Analytics software. Pyruvate and lactate were assayed using untargeted global metabolomics analysis in A549 (*H* and *I*) and Par-C5 (*J* and *K*) cells depleted of PKCδ and in control cells. *L* and *M*, Par-C5 cells depleted of PKCδ (shδ110) and control (shNT) cells were starved of glutamine for 18 h and then assayed for pyruvate and lactate using untargeted global metabolomics analysis, 0, 1, 6 and 24 h following re-addition of glutamine. Error bars in (*A* and *E*) represent SD. Statistics in *B*–*D*, *F*–*M* represent 1-way ANOVA, error bars are SEM, and ∗*p* < 0.05, ∗∗*p* < 0.01, ∗∗∗*p* < 0.001, ∗∗∗∗*p* < 0.0001. PKCδ, protein kinase Cδ.

Our data suggests that depletion of PKCδ has significant effects on pathways that regulate energy production. To further explore the effect of PKCδ on cell energetics, we used the Seahorse Mito Stress Test to measure oxidative phosphorylation in Par-C5 shNT, shδ110, and shδ680 cells. Both shδ cell lines showed a dramatic decrease in oxygen consumption (Fig. 6*A*). This reflects decreases in basal and maximal respiration (Fig. 6, *B* and *C*). Using the Seahorse Real-Time ATP Rate assay, we assessed the contribution of anaerobic glycolysis and oxidative phosphorylation to ATP production by measuring the rates of ATP production from both sources simultaneously. There was a significant shift toward increased generation of ATP from glycolysis in both shδ cell lines (Fig. 6*D*). The graphs in Figure 6, *E*–*G* show the proton efflux rate (PER) over time (glycolytic rate) and basal and compensatory glycolysis. PKCδ KD increased compensatory glycolysis in both PKCδ-depleted cell lines, while basal glycolysis was increased in shδ110 cells. Further, both steady state levels of pyruvate and lactate were increased in A549 shδPKC cells (Fig. 6, *H* and *I*), while Par-C5 cells showed a trend toward increased pyruvate but not lactate (Fig. 6, *J* and *K*). Analysis of [^13^C_6_] glucose flux in Par-C5 cells indicated that lactate (and possibly pyruvate) synthesis from glucose is reduced (Fig. 4*A*), while pyruvate and lactate production from [^13^C_5_, ^15^N_2_] glutamine are dramatically increased (Fig. 5*A*). To verify increased utilization of glutamine for pyruvate and lactate production, we glutamine starved Par-C5 cells and assayed pyruvate and lactate at 0 to 24 h after replenishment of glutamine. Both metabolites increased dramatically at 6 h after glutamine replenishment in shδ110 cells but returned to the level seen in shNT cells by 24 h (Fig. 6, *L* and *M*). We conclude that as a consequence of depletion of PKCδ, energy production shifts to a higher contribution of anaerobic glycolysis to ATP production, with parallel increase of glutamine oxidation in shδ cells.

### Metabolic reprogramming upon depletion of PKCδ contributes to radioprotection

**Figure 7:**
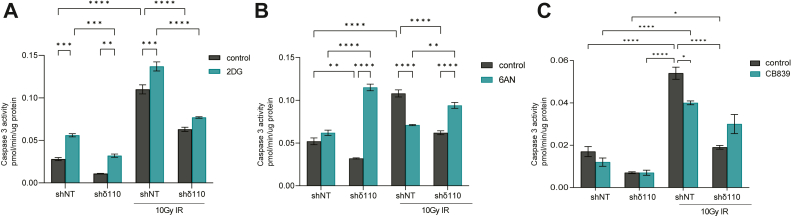
**PKCδ-depleted cells are dependent on the pentose phosphate pathway and glutamine for radioprotection.** Par-C5 cells depleted of PKCδ (shδ110) or untargeted control shRNA (shNT) were pretreated with DMSO or with indicated inhibitors for 24 h prior to treatment with 10 Gy IR. Cells were harvested 24 h following IR and measured for caspase 3 activity. *A*, 12.5 mM 2-deoxyglucose (2-DG). *B*, 50 μM 6-aminonicotinamide (6-AN), *C*, 500 μM CB-839. Statistics represent 2-way ANOVA, error bars are SEM, and ∗*p* < 0.05, ∗∗*p* < 0.01, ∗∗∗*p* < 0.001, ∗∗∗∗*p* < 0.0001. IR, irradiation; PKCδ, protein kinase Cδ.

We used pharmacological inhibitors of hexokinase and glucose-6-phosphate isomerase (2-deoxyglucose, 2DG), G6PD (6-aminonicotinamide, 6-AN), and glutaminase (CB-389) to determine if increased utilization of the PPP and/or glycolysis contributes to radioprotection in PKCδ-depleted cells. Activation of caspase in response to IR was assayed in cells pretreated with each inhibitor. Hexokinase catalyzes the conversion of glucose to G6P, the precursor of both glycolysis and the PPP. Pretreatment with 2DG resulted in increased caspase activation under all conditions (Fig. 7*A*). In contrast, both 6-AN and CB-389 had little effect on basal caspase activation in shNT Par-C5 cells (Fig. 7, *B* and *C*). However, 6-AN dramatically increased basal caspase activation in shδ110 cells (Fig. 7*B*), indicating acquired dependency on the PPP for survival. In response to IR, 6-AN significantly reduced apoptosis in shNT cells but increased apoptosis in shδ110 cells (Fig. 7*B*). Similar results were seen when irradiated cells were pretreated with CB-389 (Fig. 7*C*), although in this situation, increased caspase activation in CB839-treated shδ110 cells was only evident after IR. Taken together, these data suggest that shδ110 cells have acquired dual dependency on the PPP and glutamine for survival and that these dependencies contribute to radioprotection in the context of PKCδ depletion.

## Discussion

In this study, we demonstrate a novel role for PKCδ as a master regulator of metabolism in epithelial cells. Depletion of PKCδ reduces glucose and increases glutamine utilization for nucleotide and antioxidant synthesis, the TCA cycle, and glycolysis. In addition, PKCδ KD is associated with increased utilization of the PPP and a switch from oxidative phosphorylation to glycolysis. This suggests that loss of PKCδ rewires cells to be resistant to DNA-damaging agents, including IR, in part by increasing the production of nucleotides needed for DNA repair.

PKC isoforms, including PKCα, β, and ε, have been linked to modulation of glucose and lipid metabolism in the context of metabolic diseases (17); however, there are few studies that address the role of PKCδ in metabolic homeostasis. A proteomic analysis by Mayr et al. reported that enzymes required for glycolysis were decreased, while enzymes that regulate lipid biosynthesis were increased in hearts from PKCδ^−/−^ mice (18). Similarly, loss of PKCδ correlated with a reduction in glycolytic metabolites and an increase in fatty acid metabolites in serum from PKCδ^−/−^ mice (19). In hepatocytes, PKCδ has been linked to suppression of insulin signaling, activation of NADPH oxidase, and a concomitant increase of oxidative stress (20). Likewise, PKCδ inhibited insulin signaling and increased glucose uptake in a mice model of obesity due to leptin receptor mutation (21). A role for PKCδ in regulating insulin sensitivity and glucose tolerance suggests that loss of PKCδ function may improve the outcome of metabolic diseases. Other studies show that loss of PKCδ function *in vivo* can be protective in tissue injury models and in mouse models of neurodegenerative disease (3, 22, 23). Loss of PKCδ also facilitates protection against radiation damage *in vivo*; however, whether this involves metabolic reprogramming has not been previously addressed. It will be interesting to determine if protection against radiation injury is mechanistically similar to protection against other forms of tissue injury in the context of PKCδ depletion.

Our metabolic flux analysis in epithelial cells supports earlier data showing a role for PKCδ in glucose utilization and identifies PKCδ as an important regulator of the PPP for nucleotide and NADPH generation. At the mechanistic level, PKCδ KD increased the enzymatic activity of G6PD, the rate-limiting step of the PPP. While previous studies have shown a link between PKCδ and NADPH levels, the reported mechanism was through PKCδ-dependent activation of NADPH oxidase (24). Here, we propose that PKCδ depletion produces NADPH *via* activation of G6PD. Of note, while A549 cells show increased 6-phosphogluconate and ribose phosphate upon PKCδ depletion, the steady state level of these metabolites is not increased in Par-C5 shδ cells despite the increased activity of G6PD. This may be explained by increased utilization of 6-phosphogluconate and ribose phosphate to ultimately produce PRPP and nucleotide synthesis at a higher rate than in A549 cells; however, this hypothesis will need further confirmation. In contrast to our data which shows upregulation of GAPDH activity with PKCδ depletion, Gupte *et al* have reported that GAPDH activation by KCl is blocked by PKCδ depletion (25). At this time, we do not know the underpinnings for G6PD modulation by PKCδ; further studies will need to focus on examining whether direct (*e.g.*, phosphorylation of G6PD by PKCδ) or indirect mechanisms are at play. However, we have previously reported that PKCδ depletion increases expression of TIGAR, a positive regulator of the PPP that has been associated with chemoresistance (26, 27, 28). The connection between TIGAR, the PPP, and PKCδ merits further investigation.

Glucose tracing experiments suggest that PKCδ controls glycolysis, with PKCδ KD leading to decreased utilization of glucose for TCA metabolism. Conversely, PKCδ KD increased compensatory glycolysis while reducing oxidative respiration (both basal and maximal respiration). As a result, 60% of the ATP was produced through glycolysis in Par-C5 PKCδ KD cells. Alternatively, studies from Acin-Perez have shown that activation of PKCδ increases oxidative phosphorylation through regulation of pyruvate dehydrogenase activity, a possibility that should be addressed in the future (11). In our current studies, loss of PKCδ shifts cells to utilize glutamine in biosynthetic pathways instead of oxidative respiration. Metabolite tracing of glutamine in PKCδ KD cells revealed that labeled glutamine was utilized for synthesis of nucleotides, glutathione, and TCA intermediates. Likewise, PKCδ KD cells show an increase in labeled α-KG and increased flux of glutamine through the TCA cycle.

We have uncovered a novel role for PKCδ in metabolic reprogramming to increase nucleotide synthesis, consistent with the increased capacity of PKCδ-depleted cells for DNA repair (29). Cells can increase PPP activity to produce more nucleotides in response to DNA damage and/or and increased demand for proliferation (30, 31). Flux analysis using [^13^C_6_] glucose and [^13^C_5_,^15^N_2_] glutamine indicates that PKCδ-depleted cells increase utilization of glutamine to fuel synthesis of nucleobases and glucose for generation of ribose phosphate *via* the PPP. This includes both through increased generation of aspartate from glutamate for purine and pyrimidine synthesis and increased use of glutamine as an N donor for purine synthesis.

PKCδ-depleted cells are codependent on the PPP and increased glutamine utilization for survival after IR damage, indicating that these reprograming events contribute to radioprotection. Inhibition of hexokinase with 2DG increased cell death in both control and PKCδ KD cells, while inhibition of G6PD increased cell death in δsh110 cells but decreased cell death in control cells. This is consistent with a switch from utilization of glucose to utilization of glutamine for survival. In PKCδ KD cells, utilization of glutamine is facilitated by increased expression of glutaminase 1/2, and pretreatment with the glutaminase-1 (GLS1) inhibitor CB-839 reversed radioprotection in PKCδ-depleted Par-C5 cells. Radioresistant cells have been shown to have a high demand for glutamine, and inhibition of GLS1 can radiosensitize numerous cancer cell types (32, 33, 34). Other studies show that overexpression of GLUL promotes radiation resistance and that GLUL KD can enhance radiosensitivity (35). In contrast to our findings, overexpression of PKCδ reduced GLUL expression in glial cells (36). It is possible that epithelial and glial cells regulate divergent signaling pathways which crosstalk with PKCδ to determine GLUL levels.

Our studies show that suppression of PKCδ results in metabolic rewiring to create a radioresistant phenotype. Characteristics of this phenotype, including increased activity of the PPP and increased dependency on glutamine, are shared with many cancer cells and with cells that are chemo and radiation resistant (37, 38, 39, 40). For instance, in colon and lung cancer, inhibition of G6PD, the rate-limiting enzyme in the PPP, can reverse cisplatin resistance (40, 41). Similarly, inhibition of G6PD reversed radioprotection in PKCδ-depleted Par-C5 cells. The contribution of PKCδ to EGFR-resistant phenotypes has also been demonstrated (42, 43). It will be interesting to determine if PKCδ expression or activity in cancer cells predicts chemoresistance. Our data suggests that PKCδ may play a fundamental role in regulating radiation, and perhaps chemo-sensitivity, through changing the metabolic landscape to promote DNA repair. Further studies will be needed to understand in greater depth how these metabolic changes are regulated and their contribution to therapy-resistance phenotypes.

## Experimental procedures

### Cell culture and generation of shRNA stable KD cell lines

Par-C5 (RRID: CVCL_D695) cells have previously been described (44). A549 (RRID: CVCL_0023) cells were obtained from the CU Anschutz Cell Technologies Shared Resource and cultured in RPMI1640 (Thermo Fisher Scientific, # SH30027.01) supplemented with 10% fetal bovine serum (Sigma, #F2442) and grown in 5% CO2 humid cell culture incubator. Cell line profiling for authentication was done through the CU Anschutz Cell Technologies Shared Resource at the University of Colorado Anschutz Medical Campus. Cells used in these experiments were within 10 passages of authentication and were monitored for *mycoplasma* once a month using the Plasmotest kit from Invivogen. Par-C5 cells were stably depleted of PKCδ by transduction with lentivirus against PRKCD or a nontargeting shRNA (Cat# V3SR11242-243761456 (shδ110), Cat#V3SR11242-242319029 (shδ680), Cat#VSC11722 (shNT), Sigma-Aldrich). Human cell line A549 was stably depleted of PKCδ with the following shRNA (#TRC00010193 (sh193), #TRC00010203 (sh203), #TRCN0000196625 (sh625) or a nontargeting shRNA (shSCR), Open Biosystems). HEK-293T (RRID: CVCL_0063) cells cultured in Dulbecco’s modified Eagle’s medium/high glucose medium supplemented with 10% fetal bovine serum were used to package lentiviral particles containing shRNA following transfection with jetPRIME (Cat# 101000046, Polyplus) and lentiviral packaging vectors (45).

We obtained inhibitors for three metabolic pathways: G6PD, hexokinase, and GLS1. Specifically, we used 6-AN (Cat# A-68203) and 2-DG (Cat# D3179), Sigma-Aldrich, for G6PD and hexokinase, respectively, and telaglenastat (CB-839, Cat# S7655; Selleckchem) for GLS1. All inhibitors were added 24 h prior to 10 Gy γ-IR exposure from a cesium-137 source.

### Metabolomics

Par-C5 and A549 cells depleted of PKCδ and control cells (shNT or shSCR) were submitted to the CU Anschutz Mass Spectrometry Metabolomics Shared Resource (*Cancer Center* *Support* *Grant* *(P30CA046934))* for global and metabolic flux analysis. Global metabolomics were performed on cells grown in Par-C5 or A549 complete media. For Par-C5 flux analysis, cells were incubated in Par-C5 media lacking either glucose or glutamine for 18 h, at which time (time 0) media was changed to Par-C5 media containing either 25 mM [U-^13^C]D-glucose (Cat# 389374) or 5 mM [^13^C_5_,^15^N_2_]L-glutamine (Cat# 607983; Sigma-Aldrich). Cells were incubated in tracer media for 1, 6, or 24 h. Cells for both global and flux experiments were harvested by mechanically lifting cells off the plate, recording cell counts, and pelleting and snap freezing at - 80 °C.

Metabolites were extracted from frozen cell pellets at 2 million cells per mL by vigorous vortexing in the presence of ice cold 5:3:2 MeOH:MeCN:water (v/v/v) for 30 min at 4 °C. Following vortexing, supernatants were clarified by centrifugation (10 min, 12,000*g*, 4 °C) and an aliquot of each extract was diluted 1:1 with the aforementioned solution during transfer to autosampler vials. The resulting samples were analyzed (10 μL per injection) by ultra-high-pressure liquid chromatography coupled to mass spectrometry (Vanquish and Q Exactive, Thermo Fisher Scientific). Metabolites were resolved on a Kinetex C18 column using a 5-min gradient method as previously described (46). Following data acquisition, .raw files were converted to .mzXML using RawConverter then metabolites assigned, and peaks integrated using Maven (Princeton University) in conjunction with the KEGG database and an in-house standard library. ^13^C, ^15^N isotopic enrichment was visualized using GraphPad Prism 9.0. ^13^C_2_, ^15^N, and ^15^N_2_ metabolite peak areas were corrected for natural abundance. Quality control was assessed as using technical replicates run at beginning, end, and middle of each sequence as previously described (47).

Cell count–normalized metabolomics data was imported into MetaboAnalyst 5.0 (16) where it was log-transformed, auto-scaled, and used for subsequent PLS-DA, heat map, quantitative enrichment, and pathway topology analysis. Assessment of the quality of replicates was performed on all samples through PLS-DA for detection of outliers and clustering patterns. Construction of heatmaps involved Minkowski distance measure with average or Ward linkage clustering as indicated. Metabolite set enrichment analysis was performed using quantitative enrichment analysis, the SMPDB metabolite reference set, and a *p* < 0.05 cutoff. Enrichment ratio represents the number of observed metabolite hits divided by the number of expected metabolite hits within each pathway. Pathway impact was conducted on metabolites changed >1.2-fold change in PKCδ-depleted cells compared to shNT cells, with a significance cutoff of < 0.1. Pathway impact quantifies the number of metabolites that are present in a particular KEGG pathway following pathway enrichment (Globaltest) and pathway topology analysis (degree centrality and betweenness centrality). Pathway impact scores > 0.1 (arbitrary scale of 0–1) are shown. This means that 10% or more of the pathway metabolites vary when compared to the control.

### Immunoblot analysis

Immunoblot analysis was performed following lysis of cell pellets in JNK lysis buffer (25 mM Hepes pH 7.5, 300 mM NaCl, 1.5 mM MgCl2, 0.2 mM EDTA, 0.1% Triton X-100, 0.5 mM DTT, and 1× HALT Protease and Phosphatase Inhibitor Cocktail (Thermo Fisher Scientific)), quantification of protein concentration (DC protein assay #5000111, Bio-Rad) and separation of protein by SDS-PAGE. Proteins were transferred to polyvinylidene difluoride membranes and membranes stained with Ponceau S (Sigma-Aldrich, P3504) following transfer to confirm equal transfer and loading. Antibodies to PKCδ and β-actin were obtained from Proteintech (14188-1-AP) and Abcam (ab49900), respectively. Chemiluminescent images were obtained following incubation with Millipore Luminata Forte ECL reagent and imaging with KwikQuant Image Analyzer 5.9 (Kindle Biosciences).

### Quantitative PCR analysis of mitochondrial gene expression

mRNA gene expression was quantified using the StepOnePlus Real-Time PCR System produced by Applied Biosystems and SYBR Select Master Mix (Thermo Fisher Scientific). PrimeTime qPCR primer assays were obtained from IDT for GLS1 (Rn.PT.58.44759709), glutaminase-2 (Rn.PT.58.46148356.g), and GLUL (Rn.PT.58.8245284). The instrument automatically determined CT, and the relative expression fold change was calculated using the 2-ΔΔCT method (48). For normalization of mRNA expression levels, a panel of reference genes was used to determine the most stable normalization gene for each run. The relative expression levels were calculated as fold enrichment compared to control shNT cells (ΔΔCT). All samples were analyzed in biological triplicates, and the data is presented as mean ± SEM.

### Seahorse oxygen consumption rate analysis

The Seahorse XF96 Extracellular Flux analyzer (Agilent) was used for *in vivo* measurement of oxygen consumption rate, extracellular acidification rate, and PER rates. Mito Stress Test (Cat# 103015-100), glycolytic rate (Cat# 103344-100), and Real-Time ATP Rate (Cat# 103592-100) assays were performed according to the manufacturer's recommendations with the exception of FCCP concentration which was optimized for cell type (Par-C5, 1 μM). The Mito Stress Test assay analysis generated measurements of oxygen consumption rate attributed to basal and maximal respiration rates. The glycolytic rate assay allowed for examination of glycoPER, as well as basal and compensatory glycolysis. For all Seahorse assays, cells were plated at a previously optimized density (Par-C5–7500 cells/well), allowed to grow overnight, and analyzed on the Seahorse XF96 analyzer the next day. The Real-Time ATP Rate assay quantifies the rate of mitochondrial- and glycolytic-derived ATP production. Seahorse analytics report generators were used for all of the calculations. Experiments were designed with a minimum of four replicates per condition.

### Caspase activity assay

Caspase 3 activity was measured using the Caspase-3 Cellular Activity Assay Kit Plus (Biomol, Farmingdale, NY; BML-ALK7030001) according to manufacturer’s recommendations. This assay utilizes a colorimetric substrate, *N*-acetyl-DEVD-p-nitroaniline, that is cleaved to detect caspase 3 activity.

### G6PD activity assay

The G6PD activity was assayed using the G6PD Activity Colorimetric Assay Kit (Cat # ab102529, Abcam) according to the manufacturer’s protocols. Briefly, cells were harvested and washed in PBS prior to being lysed in JNK lysis buffer (25 mM Hepes pH 7.5, 300 mM NaCl, 1.5 mM MgCl2, 0.2 mM EDTA, 0.1% Triton X-100, 0.5 mM DTT, and 1× HALT Protease and Phosphatase Inhibitor Cocktail (Thermo Fisher Scientific)). Protein concentration was assayed using the BCA kit (Bio-Rad). An equal amount of protein from each cell line was used to perform the assay. Absorbance at OD 450 nm was measured on a microplate reader (SpectraMax190, Molecular Devices) in kinetic mode for 30 min at 37 °C. The G6PD activity in biological triplicates was assayed at 5 min and 30 min.

### NADPH/NADP+ assay

The NADPH/NADP+ ratio was measured using the NADPH/NADP+ Fluorometric Assay Kit (Cat # ab176724, Abcam) according to the manufacturer’s protocols. An equal number of cells from each cell line was used to perform the assay. Briefly, Par-C5 shNT, shδ110, and shδ680 cells were harvested and washed in PBS and lysed in NADPH/NADP+ lysis buffer. The lysate was collected and used to perform the assay. Fluorescence intensity of biological triplicates was measured on a microplate reader (Synergy2, Biotek) at a 560 nm excitation wavelength and a 590 nm emission wavelength.

### Statistical analysis

Unless otherwise indicated, figures depict representative experiments, utilizing triplicate samples (Caspase-3 activity, G6PD, and NADPH/NADP+) that were conducted a minimum of three times, and error bars denote SEM. Statistical analysis was performed either within MetaboAnalyst 5.0 (www.metaboanalyst.ca) software or using GraphPad Prism 9 software (www.graphpad.com), utilizing student’s *t* test or a one-way or two-way ANOVA as indicated (significance level of 0.05) with Dunnett's multiple comparisons (#*p* < 0.10; ∗*p* < 0.05; ∗∗*p* < 0.01; ∗∗∗*p* < 0.001; ∗∗∗∗*p* < 0.0001).

## Data availability

All data are available in the main text or in the Supporting Information section. Mass spectrometry data is available upon request from Dr D’Alessandro (angelo.dalessandro@cuanschutz.edu).

## Supporting information

This article contains supporting information.

## Conflict of interest

The authors declare that they have no conflicts of interest with the contents of this article.
