# Supplementary material for: Metabolic reprogramming contributes to radioprotection by protein kinase Cδ
Source: J Biol Chem. 2023 Aug 21;299(10):105186. doi: 10.1016/j.jbc.2023.105186 (PMC10519828; doi:10.1016/j.jbc.2023.105186)
Supplement: Supporting information [file mmc1.pdf]

Metabolic reprogramming contributes to radioprotection by protein kinase Cδ

Angela M. Ohm[1], Trisiani Affandi[1], Julie A. Reisz[2], M. Cecilia Caino[3], Angelo D'Alessandro[2], and Mary E. Reyland[1]*

[1]Department of Craniofacial Biology, School of Dental Medicine, University of Colorado Anschutz Medical Campus, Aurora, CO 80045, USA

[2] Department of Biochemistry and Molecular Genetics, University of Colorado Anschutz Medical Campus, Aurora, CO 80045, USA

[3] Department of Pharmacology, School of Medicine, University of Colorado Anschutz Medical Campus, Aurora, CO 80045, USA

Supporting Information Figure S1: Metabolic profiling of Par-C5 shδ680 cells.

Supporting Information Figure S2: Metabolic profiling of A549 cells depleted of PKCδ.

Supporting Information Figure S3: Nucleotides and PPP metabolites in PKCδ depleted A549 cells.

Supporting Information Figure S4: Nucleotide total metabolite plots from glucose and glutamine tracing.

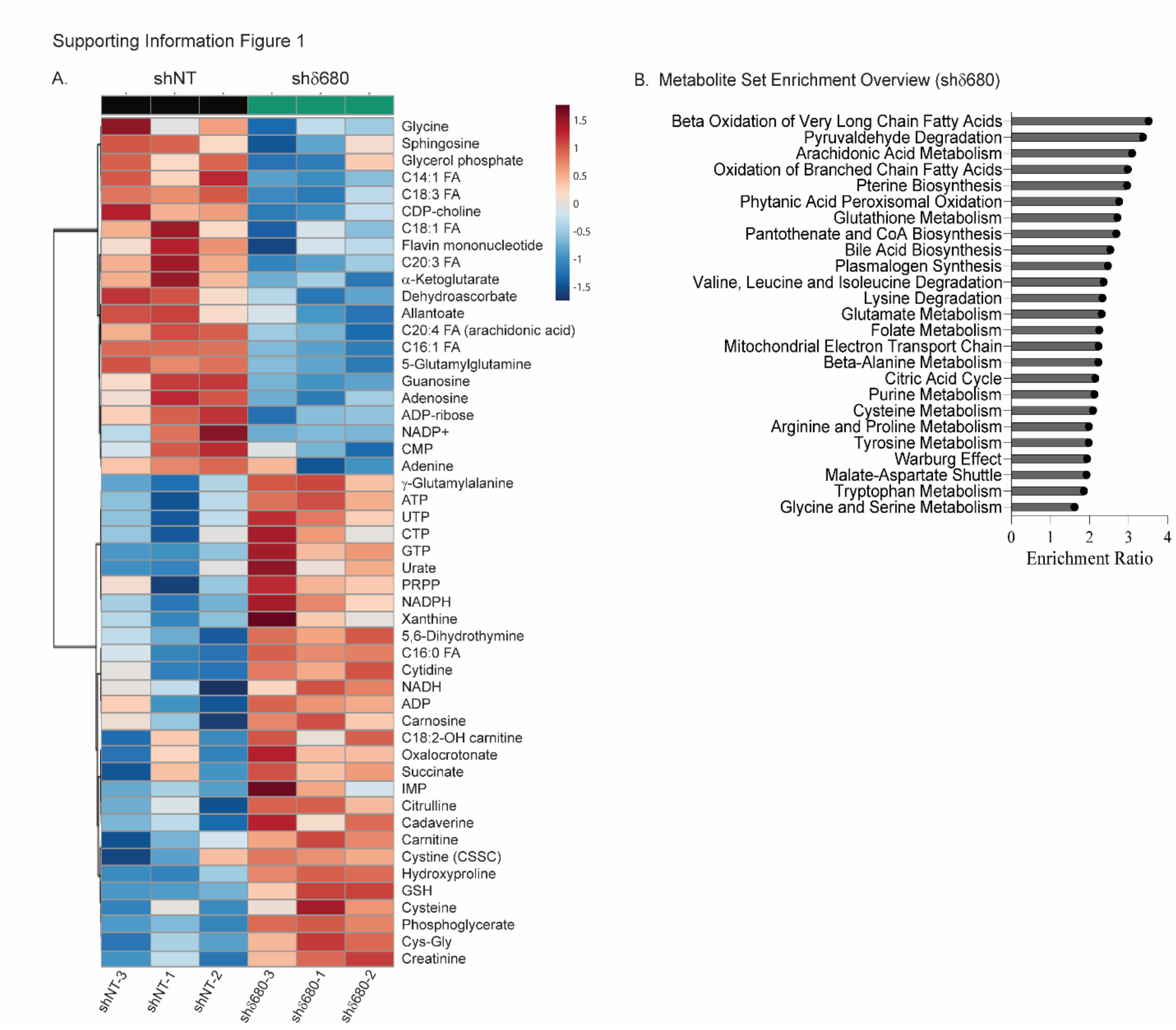

Supporting Information Figure S1: Metabolic profiling of Par-C5 shδ680 cells. Untargeted metabolomics was performed on triplicate biological samples of Par-C5 cells depleted of PKCδ (shδ680) or untargeted control shRNA (shNT). A, Heat map with hierarchical clustering of the top 50 significant metabolites in Par-C5 shNT and shδ680 cells following student's t-test analysis ($\alpha = 0.05$). B, Enrichment analysis of shδ680 cells as compared to shNT cells. The top 26 metabolite groups from enrichment over-representation analysis are shown in the bar chart ($p < 0.05$). Enrichment ratio represents the number of observed metabolite hits divided by the number of expected metabolite hits within each pathway.

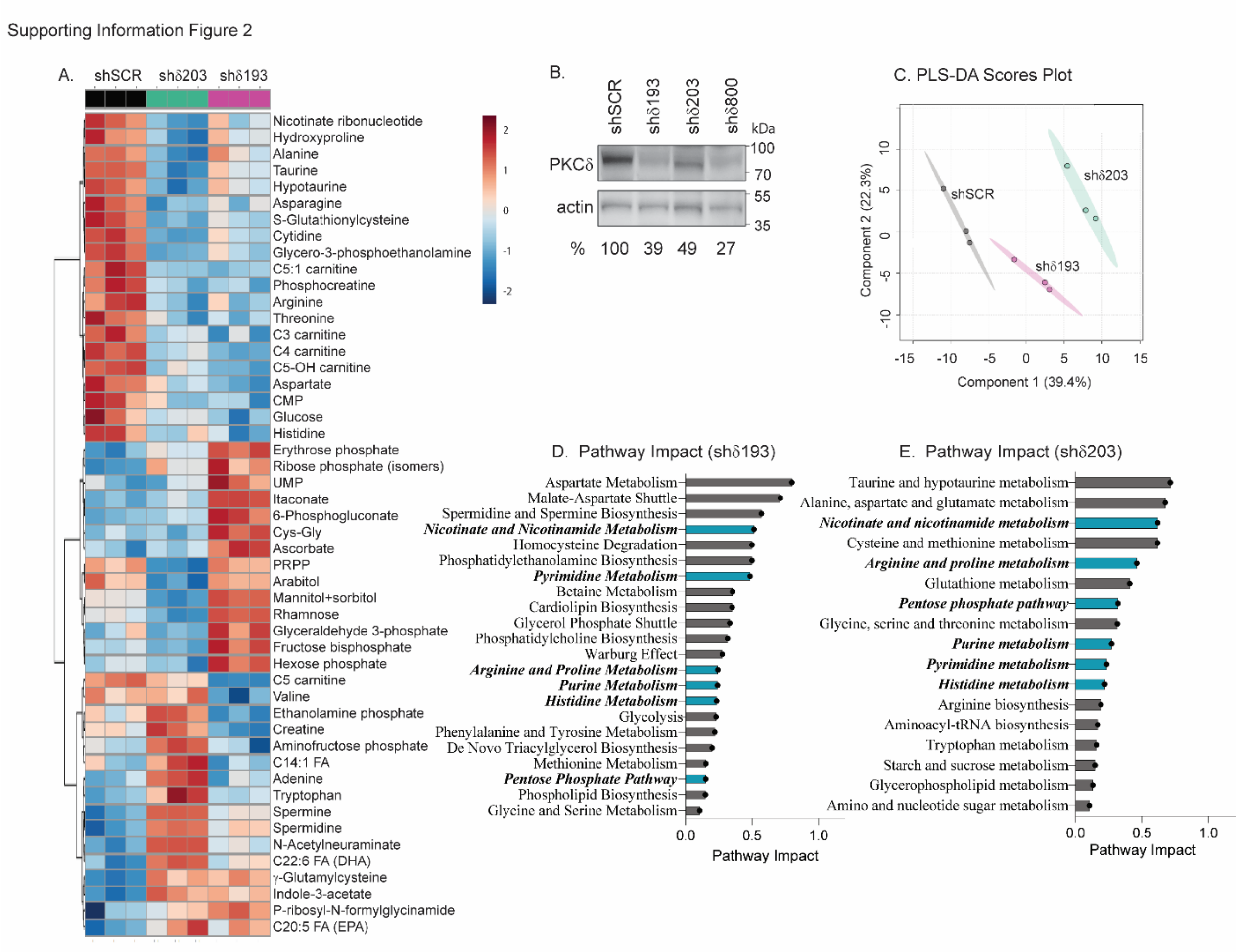

Supporting Information Figure S2: Metabolic profiling of A549 cells depleted of PKCδ. Untargeted metabolomics was performed on triplicate biological samples of A549 cells depleted of PKCδ (shδ193 and shδ203) or untargeted control shRNA (shSCR). A, Top 50 significant metabolites in shSCR, shδ193 and shδ203 depleted cells are shown following student's t-test analysis ($\alpha = 0.05$). Heatmap represents hierarchical clustering with Minkowski distance measure and Ward linkage clustering. B, Immunoblot showing the depletion of PKCδ with three different human shRNA constructs. Densitometry quantifying the percent of PKCδ expression, as compared to shSCR and normalized to actin, is shown below the blots. C, Partial least squares-discriminant analysis (PLS-DA) scores plot of metabolomics data. Shaded ovals represent 95% confidence intervals. D-E, Pathway impact was determined with untargeted global metabolomics of A549 cells depleted of PKCδ shδ193 (D) and shδ203 (E) cells using MetaboAnalyst (see Experimental Procedures). Shown are pathways with greater than 0.10 pathway impact score. Italicized and bolded pathways are those shared between shδ193 and shδ203 depleted cells.

Supporting Information Figure S3

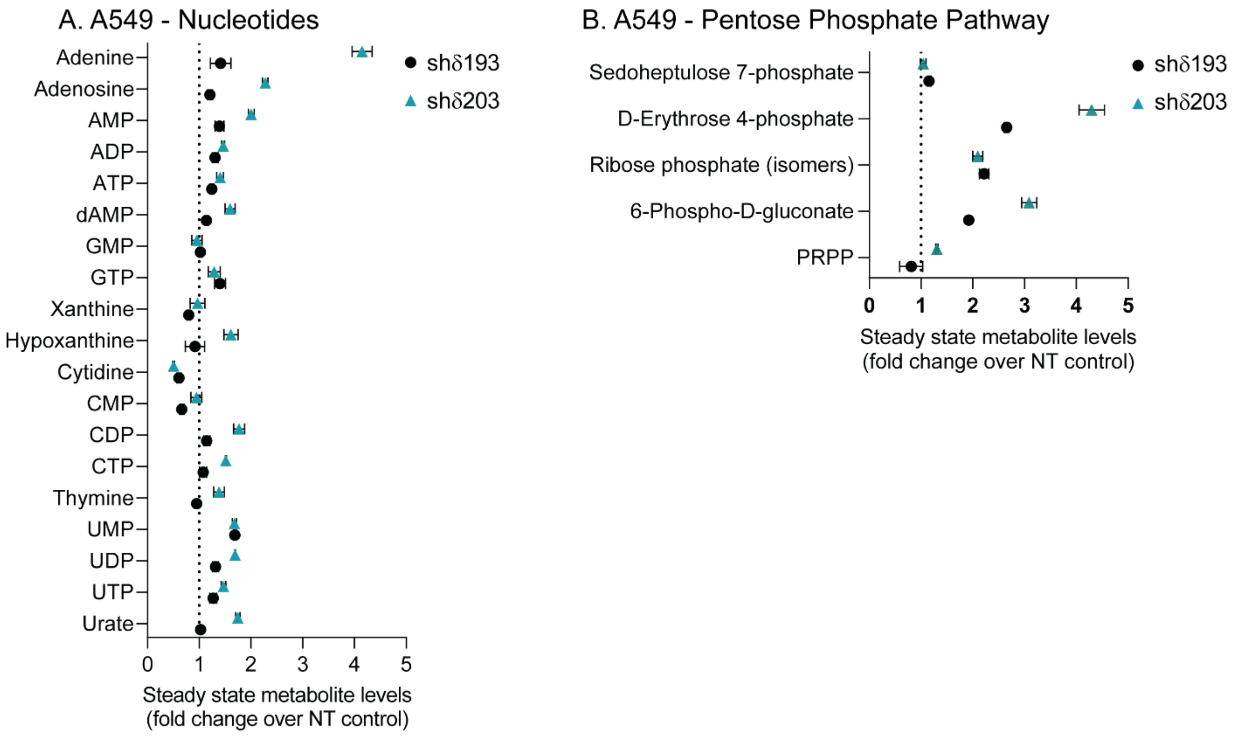

Supporting Information Figure S3: Nucleotides and PPP metabolites in PKCδ depleted A549 cells. A and B, Individual metabolite measurements from top pathways identified in A549 PKCδ depleted cells (shδ193 or shδ203) from Supplemental Figure 2. Data is normalized to shSCR = 1 and expressed as fold change over shSCR.

Supporting Information Figure S4

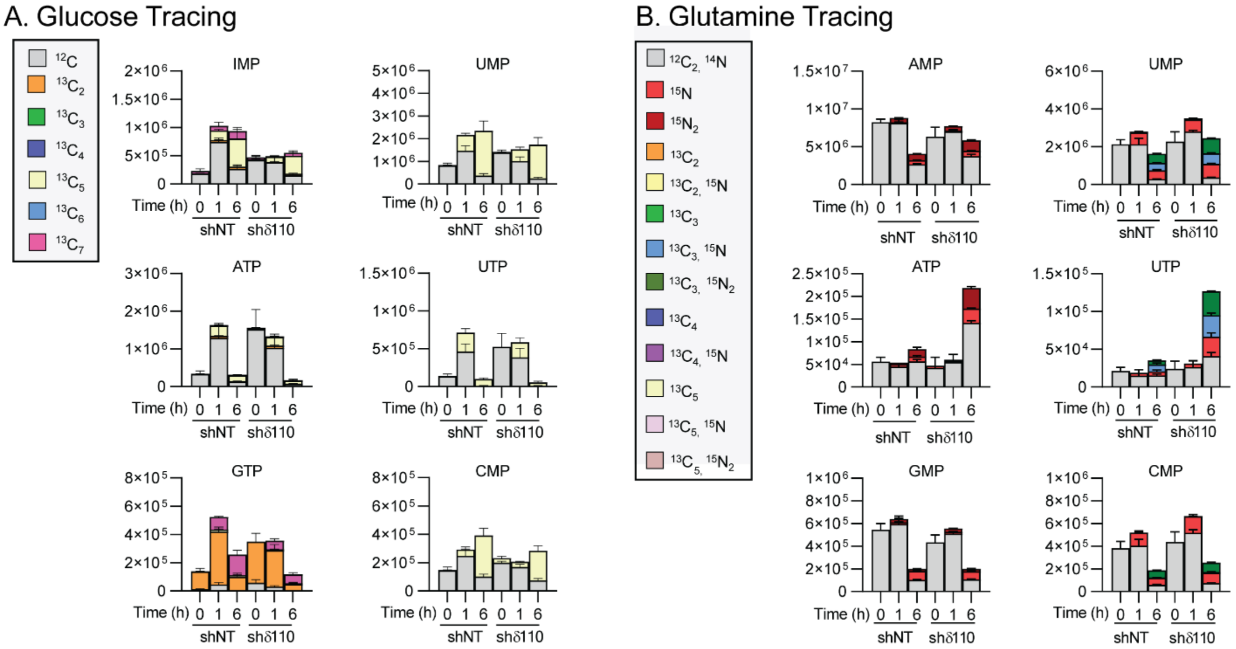

Supporting Information Figure S4: Nucleotide total metabolite plots from glucose and glutamine stable isotope tracing analysis. A, Par-C5 cells were labelled with [U-^{13}C]Glucose for 0, 1, and 6 hours. B, Par-C5 cells were labelled with [$^{13}C_5{}^{15}N_2$]Glutamine for 0, 1, and 6 hours. Stacked bar graphs identify unlabeled metabolites (gray) and enriched isotopologues; data is presented as mean +/- SEM.
